# Deposition of carbon from methane on manganese sources

**DOI:** 10.1038/s41598-023-29269-6

**Published:** 2023-02-07

**Authors:** Halvor Dalaker, Jonas E. Gjøvik, Eli Ringdalen

**Affiliations:** SINTEF, Trondheim, Norway

**Keywords:** Metals and alloys, Process chemistry

## Abstract

Carbon has been deposited on HCFeMn slag from methane-containing gas with and without CO_2_, creating C-MnO composites and giving a hydrogen-rich off-gas as a by-product. The maximum deposited amount corresponds to 38 ± 6% of the carbon required for reduction of all manganese in the slag to metallic Mn. This was achieved at 1100 °C with a H_2_-concentration in the off gas of 76%. Temperature was an important parameter. At 790 °C, no deposited carbon was detected, at temperatures ≥ 1000 °C, deposition increased with temperature. A lower gas-flow leads to more methane decomposition. Experiments with CO_2_ in the process gas gave less deposited carbon than other experiments. This could be caused by dilution of methane or chemical reactions involving CO_2_, or a combination. Investigations of fines formation indicate that the deposited carbon sticks well to the HCFeMn-slag, and would not fall off easily during transport and handling. This demonstrates that biogas can potentially be a non-fossil source of carbon in manganese production.

## Introduction

As in any other industry, to reach the climate goals of the Paris Agreement, the manganese industry must reduce its fossil CO_2_ emissions. Mn-alloys are mainly produced in submerged arc furnaces (SAF) by carbothermic reduction of oxidic raw materials. Since carbon in Mn-alloy production is used as a reductant to remove oxygen and not as an energy source it is not easily replaced. One option is to replace fossil carbon with biologically sourced carbon, which will reduce fossil (but not total) CO_2_ emissions.

Several studies have looked at using solid biocarbon in the form of charcoal in manganese production^1–3^. There are challenges related to the mechanical strength and low density^1,4^, and charcoal also reacts more strongly towards CO_2_ than metallurgical coke^5^. This causes carbon to be consumed outside the reaction zone, increasing carbon consumption and CO_2_ emissions.

Biogas, produced from the digestion of organic matter, is also a potential source of non-fossil carbon, in the form of methane gas, CH_4_. While some studies show interesting results of using methane directly as the reducing agent in producing manganese^6^, this has so far not been industrialised. A different option is to convert the methane in biogas into solid carbon through thermal cracking.

**Figure 1:**
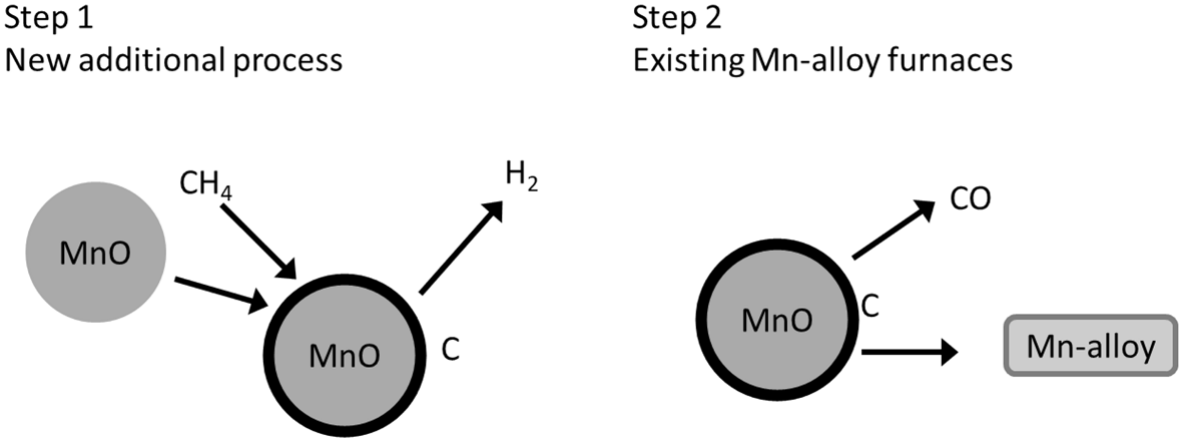
Principle sketch of the idea behind the current work. A two-step process is imagined. Step 1 is a new process: the deposition of carbon on MnO from methane, with hydrogen as a by-product. Step two is to use the produced composites in existing furnaces, in a process very similar to today's.

The main idea behind the current work is to treat Mn-ore with methane from biogas at high temperatures where the methane decomposes to carbon and hydrogen. If the carbon can be made to deposit onto the Mn-ore, to stick there during transport, and to act as a reductant in Mn-furnaces, the carbon in this composite material can be used as a non-fossil reducing agent by introducing a two-step process as shown in Fig. 1. The first step will be the deposition of carbon from methane onto manganese ore in a separate unit; the second step will be to use the produced composite material to produce manganese in a process very similar to that being used today. The hydrogen resulting from the decomposition step could be a valuable by-product.

The primary goal of the current work is to use thermal cracking of methane to demonstrate that it is possible to deposit solid carbon from biogas on manganese ore and that the carbon sticks to the manganese ore, in other words to demonstrate the feasibility of step 1 of Fig. 1.

Methane decomposes after the overall reaction:

$${\text{CH}}_{4} \to {\text{C}}_{{\text{(s)}}} + 2{\text{H}}_{2}$$

This reaction is thermodynamically favourable at temperatures above approximately 550 °C, but methane can exist in a metastable condition at much higher temperatures. In order to break the stable C–H bonds and promote the decomposition, either higher temperatures or the use of a catalyst is needed.

If the decomposition of methane occurs on a surface, the generally agreed mechanism is an adsorption of CH_4_ followed by progressive dissociation of H atoms, their release to the gas-phase as H_2_ molecules, and eventually the accumulation of carbon atoms^7^:

$${\text{CH}}_{{{4}({\text{g}})}} \to {\text{CH}}_{{{4}({\text{ad}})}} \to {\text{CH}}_{{{3}({\text{ad}})}} + {\text{H}}_{{({\text{ad}})}} \to {\text{CH}}_{{{2}({\text{ad}})}} + {\text{2H}}_{{({\text{ad}})}} \to {\text{CH}}_{{({\text{ad}})}} + {\text{3H}}_{{({\text{ad}})}} \to {\text{C}}_{{({\text{ad}})}} + {\text{2H}}_{{{2}({\text{ad}})}} \to {\text{C}}_{{({\text{s}})}} + {\text{2H}}_{{{2}({\text{g}})}}$$

It is also possible that the decomposition is homogeneous in nature and takes place in the gas-phase. Several mechanisms have been proposed, but generally start with reactions of methane into two-carbon molecules like acetylene, and also typically involve cyclical molecules as an important intermediary. An example can be found in^8^, where benzene is the first cyclical molecule which transforms into naphthalene and later three- and four-ring aromatics, eventually ending up as carbon.

The precise reaction scheme between methane and solid carbon has not been the focus of the present study.

Without cleaning/upgrading, biogas contains large quantities of CO_2_, in addition to sulphur containing species and other impurities^9,10^. As sulphur is detrimental in the steels in which the manganese alloys will be used, sulphur-removal will probably always necessary. But if the biogas could be utilised without CO_2_ removal, this would obviously be a benefit. Therefore, both CH_4_ and CH_4_-CO_2_ mixtures are examined as carbon sources in the current work. At the outset, it is expected that CO_2_ could interact with the carbon deposition experiments in several ways, the first is to consume deposited carbon by the Boudouard reaction:

$${\text{C}}_{{({\text{s}})}} + {\text{ CO}}_{{2}} \to {\text{2CO}}$$

Another is to consume produced hydrogen by the water–gas-shift reaction:

$${\text{H}}_{{2}} + {\text{ CO}}_{{2}} \to {\text{H}}_{{2}} {\text{O}}_{{({\text{g}})}} + {\text{ CO}}$$

Both of these reactions would be detrimental, but it is also possible that CO_2_ could have positive effects, as CO_2_ can also shift the methane decomposition equilibrium towards more deposited carbon. As an example, the following reaction is thermodynamically favourable above 503.9 °C, according to calculations in FactSage^11^:

$$\raise.5ex\hbox{$\scriptstyle 3$}\kern-.1em/ \kern-.15em\lower.25ex\hbox{$\scriptstyle 3$} {\text{ CH}}_{{4}} + \, \raise.5ex\hbox{$\scriptstyle 1$}\kern-.1em/ \kern-.15em\lower.25ex\hbox{$\scriptstyle 4$} {\text{ CO}}_{{2}} \to {\text{H}}_{{2}} + \, \raise.5ex\hbox{$\scriptstyle 1$}\kern-.1em/ \kern-.15em\lower.25ex\hbox{$\scriptstyle 2$} {\text{ H}}_{{2}} {\text{O }} + {\text{ C}}$$

It is thus not clear from the outset what overall effect CO_2_ will have. A secondary objective of the current work is thus to investigate the effect of CO_2_ on the decomposition of methane.

The catalytic decomposition of methane has been studied both from the point of view of hydrogen production^12^ and for production of carbon nanomaterials^7^. Moreover, the catalytic reaction between CO_2_ and methane has been extensively studied in syngas production, as so called dry-reforming of methane^13^:

$${\text{CH}}_{{4}} + {\text{ CO}}_{{2}} \to {\text{2H}}_{{2}} + {\text{ 2CO}}$$

As the use of a catalyst would add both complexity and cost to the process, it is desirable to avoid adding a catalyst if possible. However, the manganese ore provides surfaces on which the reactions can take place, and so catalytic effects may nevertheless play a role. Manganese oxides are catalytically active towards CO^14^ and CO_2_ when combined with other materials such as Cu^15^ and Co_3_O_4_^16^.

Manganese sources also contain iron. If the manganese source is a pre-reduced ore, or a MnO-containing slag, then the iron oxides will have been reduced to metallic iron. If the manganese source is untreated ore, iron would initially be in the form of oxides, but in a process such as the one proposed in the current work these could be reduced to the metallic state by methane, hydrogen, or deposited carbon. As metallic iron can catalyse methane decomposition^17^, this could play a role in the initial decomposition phase.

Different catalysts will produce different carbon products under different conditions. Metallic catalysts can produce carbon filaments, or carbon nano-tubes, for example^18^. Carbon-based catalysts on the other hand, tend to produce carbon black or layered carbon^18^. For use in ferromanganese production, in order to limit reactivity towards CO_2_, the most porous structures are unwanted.

## Experimental setup and method

The manganese source used was high-carbon ferromanganese (HCFeMn) slag of industrial origin. It was chosen because in this raw material the only manganese oxide present is MnO, as opposed to various ores that also contain higher order oxides like MnO_2_. The higher order oxides could provide oxygen for reaction with the deposited carbon, making interpretation of the results harder. Another result of using HCFeMn-slag is that all of the iron oxides in the original manganese ore will have been reduced to metallic iron. Most if it was collected as metal product during ferromanganese production, but the slag still contains around 1% iron, found as metal droplets captured within the slag.

The gases used were 99.999% Argon 5.0, scientific 99.9992% CO_2_, and 99.5% CH_4_ 2.5. As process gas, two different gas mixtures were used: 90%CH_4_–10%Ar, and 63%CH_4_–27%CO_2_–10%Ar.

**Figure 2:**
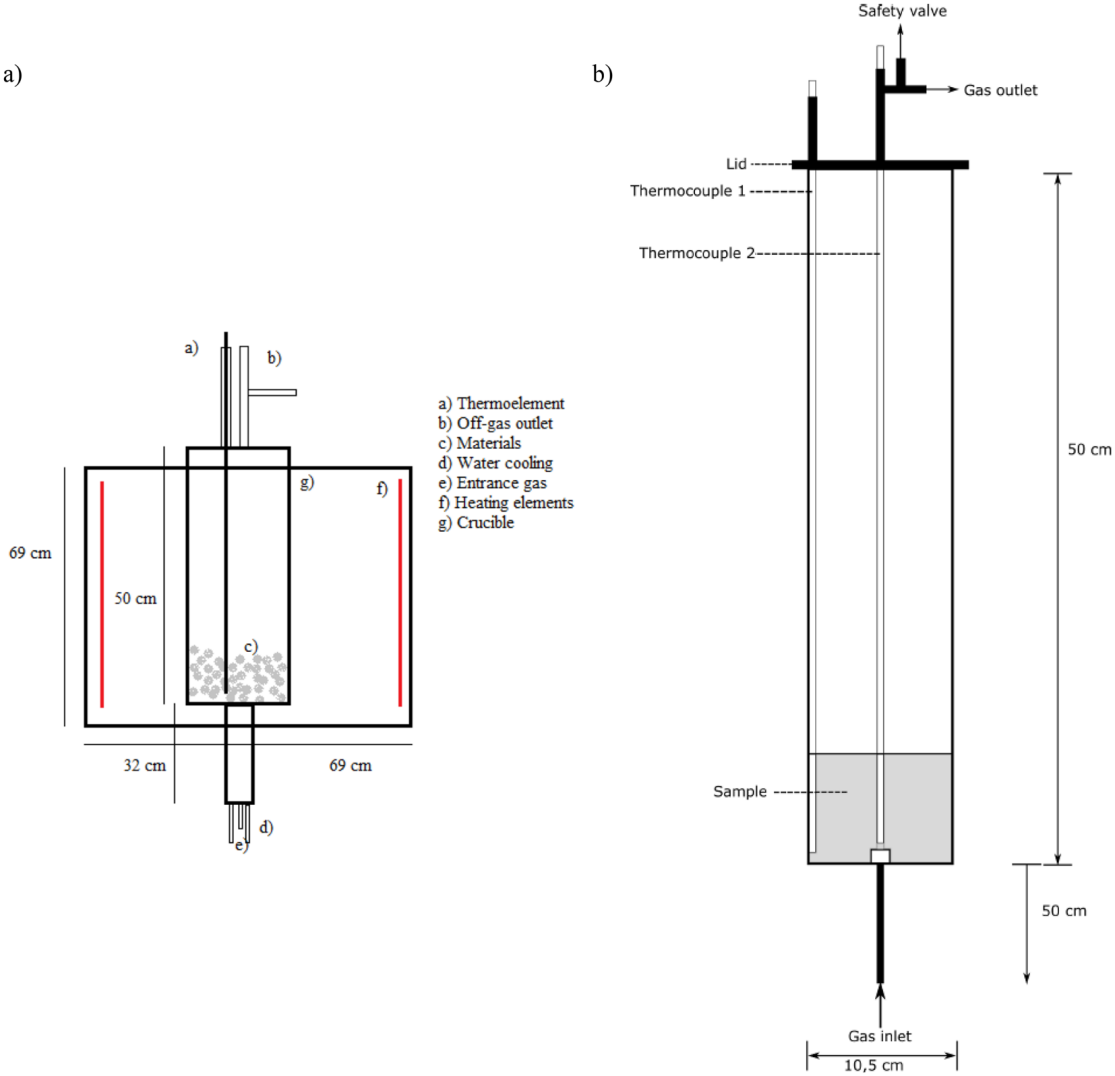
Sketch of the experimental setup. (**a**) Sketch of the whole furnace chamber^19^, while (**b**) shows the crucible in more detail.

Experiments were run in a resistance heated furnace. The furnace and crucible are illustrated in Fig. 2. In each experiment, 2000 g of HCFeMn slag was screened to sizes of 5–10 mm. This material was charged in a steel crucible which in turn was placed inside a graphite crucible on top of an inlet gas dispersing plug. Process gases were mixed with mass flow controllers (5 SLPM and 100 SLPM) and purged through the bottom of the furnace into the bottom of the crucible through the dispersing plug. The gas-inlet was water-cooled to prevent methane decomposition leading to carbon deposition and clogging of the inlet. The gas travelled into the crucible through the gas dispersing plug and into the material as shown in Fig. 2b. The off-gas exited at the top of the furnace where it was analysed by a micro gas chromatograph (Micro GC) for CH_4_, CO, CO_2_, H_2_, N_2_ and O_2_.

The total outgoing gas flow, the concentration of water vapour in the off-gas, and the net rate of formation of solid carbon were not measured, but can be calculated from known quantities. Since the total gas-flow and -composition into the furnace was known, and disregarding higher order hydrocarbons like C_2_H_2_ (which according to thermodynamic equilibrium should be present at levels orders of magnitude below 1%), the hydrogen- carbon- and oxygen mass balances give three equations with three unknowns:

$${\mathrm{\varphi }}_{0}\cdot \left({4\left[{\mathrm{CH}}_{4}\right]}_{0}+2{\left[{\mathrm{H}}_{2}\right]}_{0}\right)={\mathrm{\varphi }}_{1}\cdot \left(4 {\left[{\mathrm{CH}}_{4}\right]}_{1}+2{\left[{\mathrm{H}}_{2}\right]}_{1}+2{\left[{\mathrm{H}}_{2}\mathrm{O}\right]}_{1}\right)$$

$${\mathrm{\varphi }}_{0}\cdot {\left[2{\mathrm{CO}}_{2}\right]}_{0}={\mathrm{\varphi }}_{1}\cdot\left( {\left[\mathrm{CO}\right]}_{1}+{\left[{\mathrm{H}}_{2}\mathrm{O}\right]}_{1}+2{\left[{\mathrm{CO}}_{2}\right]}_{1}\right)$$

$${\mathrm{\varphi }}_{0}\cdot \left({\left[{\mathrm{CH}}_{4}\right]}_{0}+{\left[{\mathrm{CO}}_{2}\right]}_{0}\right)={\mathrm{\varphi }}_{1}\cdot \left( {\left[{\mathrm{CH}}_{4}\right]}_{1}+{\left[{\mathrm{CO}}_{2}\right]}_{1}+{\left[\mathrm{CO}\right]}_{1}\right)+{\left\{{\mathrm{C}}_{\left(\mathrm{s}\right)}\right\}}_{1}$$

Here a subscript "0" denotes incoming, a subscript "1" denotes outgoing, [X] denotes the concentration (moles/l) of species X, $\mathrm{\varphi }$ denotes total gas-flow (L/min), and {C_(s)_} denotes the net rate of solid carbon formation (mol/min). Solving these equations gives the unknowns ${\mathrm{\varphi }}_{1}$, [H_2_O]_1_ and {C_(s)_}_1_, and since argon is inert, the outgoing argon-concentration is simply the known incoming concentration scaled by the change in total gas flow. Multiplying{C_(s)_}_1_ by the holding time gives the net solid carbon formed during each experiment. It should be stressed that the net solid carbon *formed* does not equate to the amount of carbon *deposited* on the HCFeMn slag*.* Some of the formed carbon can for example deposit on the crucible wall or be carried off as fine dust.

The temperature of the furnace was controlled by an S-type thermocouple placed close to the heating elements. The temperature was also measured inside of the charge, indicated by thermocouple 1 and thermocouple 2 in Fig. 2b. The sample was heated to 200 °C at 13 °C/min, and then to the designated temperature at 3 °C/min while flushing with Ar at 5L/min. Once the temperature had stabilised at the target temperature, argon was switched off, the process gas was switched on, and the sample held in stable conditions for 2 h. After the 2-h holding period, the process gas and furnace power were switched off, and the sample was purged with Ar at 5 L/min until cool.

The samples were weighed before and after the experiment, and carbon content in the material measured by combustion IR spectroscopy. Additionally, the samples were imaged by conventional camera and by back-scatter imaging in an Electron Probe Micro Analyser (EPMA). The samples also underwent an abrasion test. An abrasion test is an indication of how well the deposited carbon will stick to the Mn-material during handling after it has been deposited. This test involved sieving the material at 5 mm and 500 µm before and after 30 min in a Hanover drum at 40 rpm and comparing the different size fractions.

Three different temperatures, two different gas compositions and two different total gas flows were investigated across 15 total experiments, shown in Table 1.

**Table 1:** Overview of all experiments.

Atmosphere	Exp. series #	Exp. #	Temp (°C)	Total flow rate (L/min)	Flow CO_2_ (L/min)	Flow CH_4_ (L/min)	Flow Ar (L/min)
27% CO_2_ + 63% CH_4_ + 10%Ar	1	1	790	2.35	0.63	1.48	0.24
90% CH4 + 10% Ar	1	2	790	2.35		2.12	0.24
90% CH_4_ + 10% Ar	1	3	790	4.7		4.23	0.47
27% CO_2_ + 63% CH_4_ + 10%Ar	1	4	1000	2.35	0.63	1.48	0.24
27% CO_2_ + 63% CH_4_ + 10%Ar	1	5	1000	2.35	0.63	1.48	0.24
90% CH_4_ + 10% Ar	1	6	1000	2.35		2.12	0.24
90% CH_4_ + 10% Ar	1	7	1000	2.35		2.12	0.47
27% CO_2_ + 63% CH_4_ + 10%Ar	1	8	1000	4.7	1.27	2.96	0.47
27% CO_2_ + 63% CH_4_ + 10%Ar	1	9	1000	4.7	1.27	2.96	0.47
90% CH_4_ + 10% Ar	1	10	1000	4.7		4.23	0.47
27% CO_2_ + 63% CH_4_ + 10%Ar	1	11	1100	2.35	0.63	1.48	0.24
27% CO_2_ + 63% CH_4_ + 10%Ar	1	12	1100	2.35	0.63	1.48	0.24
90% CH_4_ + 10% Ar	2	13	1100	2.35		2.12	0.24
90% CH_4_ + 10% Ar	2	14	1100	2.35		2.12	0.24
90% CH_4_ + 10% Ar	2	15	1100	2.35		2.12	0.24

As mentioned above, it is possible that CO_2_ can influence the methane decomposition by reacting with the methane. It is also possible that the kinetics of these reactions are more favourable at low temperatures than thermal decomposition of methane, which could open up the possibility of carbon production at lower temperatures than those typically associated with thermal decomposition of methane. For this reason, the study included experiments as low as 790 °C, to check if the presence of CO_2_ could help promote methane decomposition at such low temperatures.

## Results and discussion

If carbon from methane is to be used in Mn-production as intended by the current scheme, three conditions need to be fulfilled:

1. The methane needs to decompose into carbon and hydrogen
2. The carbon formed needs to deposit onto the HCFeMn slag in appreciable amounts
3. The carbon needs to stick to the surface of the HCFeMn and not easily fall off, to be able to withstand transport etc.

For optimisation and scale-up of the process, it is also useful to know the effect of different parameters on the deposition rate. The following subsections presents the results with these issues in mind.

### Methane decomposition

The off-gas measurements are presented in Table 2, averaged across the 2-h holding period during which the samples were exposed to process gas. The values for water content in off-gas, total outgoing gas flow and amount of carbon produced are also included in Table 2, calculated as described in the methodology section. Also included is the difference in incoming and outgoing methane, ΔCH_4_.

**Table 2:** Measured (µ-GC) and calculated (from mass balance) composition of off-gas and amount of solid carbon formed for all experiments.

Exp	Incoming gas			T (°C)	Flow-rate (L/min)	Outgoing gas composition						Net solid C formed		ΔCH_4_ (mol)
						Measured averages				Calculated		Calculated		
	CO_2_ (%)	CH_4_ (%)	Ar (%)			CO (%)	CO_2_ (%)	H_2_ (%)	CH_4_ (%)	H_2_O (%)	Ar (%)	(g)	C_stoich_ (%)	
1	27	63	10	790	2.35	3	28	0	66	−2	6	−3.8	−3	−0.1
2	0	90	10	790	2.35	0	0	1	100	0	7	0.3	0	0.0
3^a^	0	90	10	790	4.7	0	0	0	0	0	10	0	0	0.0
4	27	63	10	1000	2.35	27	5	22	36	5	9	4.5	3	2.0
5	27	63	10	1000	2.35	30	3	24	36	7	8	7.1	5	2.2
6	0	90	10	1000	2.35	0	0	38	58	0	9	30.5	22	2.5
7	0	90	10	1000	2.35	1	1	34	52	0	9	31	22	2.6
8	27	63	10	1000	4.7	18	8	12	37	3	8	-0.8	−1	2.5
9	27	63	10	1000	4.7	25	7	18	41	8	8	15.6	11	3.5
10	0	90	10	1000	4.7	1	0	27	68	0	9	40.9	29	3.4
11	27	63	10	1100	2.35	31	0	43	18	3	11	18.3	13	4.1
12	27	63	10	1100	2.35	28	0	40	14	1	3	16.9	12	4.3
13	0	90	10	1100	2.35	1	0	82	29	0	8	73.6	52	6.1
14	0	90	10	1100	2.35	1	0	80	29	0	8	71.8	51	6.0
15	0	90	10	1100	2.35	1	0	67	42	0	8	55.8	39	4.6

C_stoich_ means percentage of carbon necessary for self-reduction of all MnO (see text). Note that carbon *formed* does not equate to carbon *deposited*. ΔCH_4_ is the difference, in mol, between incoming and outgoing methane.

^a^In Experiment 3, an error occurred which gave no signal to the off-gas measurements.

Some of the values for calculated amount of carbon formed are negative. This means that the mass balance, using the available data, indicates that carbon was *removed* from the samples. Since no free carbon is available in the sample prior to the experiments, this is an unphysical result and is a result of the uncertainties of the gas measurements.

The (dried) slag contains 42% MnO. That means that for conversion of all MnO in a 2000 g slag sample to Mn after the reaction MnO + C = Mn + CO, 142.1 g of carbon is required. In the following discussion, "*C*_*stoich*_" is introduced to mean: the amount of carbon deposited relative to the amount required for stoichiometric conversion of all MnO (in other words, relative to 142.1 g in the current work). *C*_*stocih*_ is used not only when referring to carbon specifically deposited on HCFeMn slag, but also for example when describing the total amount of carbon formed in the system, such as in Table 2.

**Figure 3:**
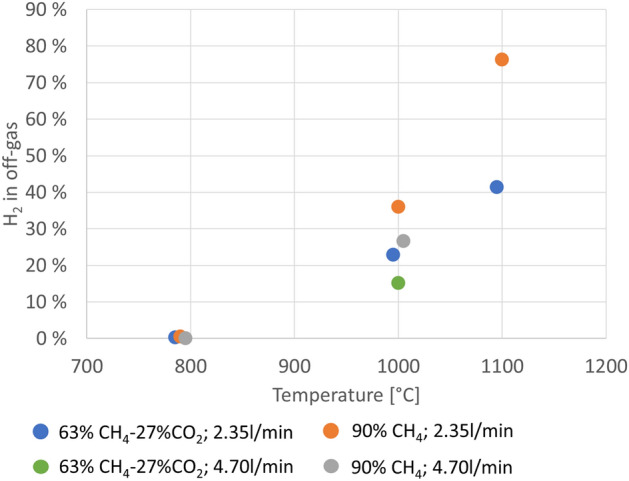
Hydrogen content in the off gas of all experiments, plotted versus temperature. For repeat experiments, each data point represents an average of all experiments with identical experimental parameters. The data points at common temperatures have been spaced out along the x-axis to improve readability. All data points have been recorded at T = 790 °C, 1000 °C or 1100 °C, respectively.

In the experiments at 790 °C, no hydrogen was detected in the off-gas, so no methane decomposition took place. This means that the presence of CO_2_ was not sufficient to drive deposition of carbon at lower temperatures. In all other experiments hydrogen was detected in the off-gas, indicating that cracking did in fact take place. Figure 3 below shows the hydrogen levels averaged across experiments with identical experimental parameters. For data points with the same colour, the only different experimental parameter was temperature. For two sets of parameters (90% CH_4_, 2.35 L/min; 63%CH_4_–27%CO_2_, 2.35 L/min) there is significant hydrogen evolution at two different temperatures (1000 °C and 1100 °C) with all other parameters the same. In both cases the amount of hydrogen is much higher at 1100 °C than at 1000 °C. The amount of data is too limited to quantify the effect, but this indicates that temperature is a very important parameter, and that methane decomposition increases with temperature.

For two sets of parameters (63%CH_4_–27%CO_2_, 1000 °C; 90%CH_4_, 1000 °C) all parameters are the same except the total gas flow. In both cases the amount of hydrogen, and thus the degree of methane decomposition, is higher in the case of a lower total gas flow.

In experiments with CO_2_ in the process gas, this was converted into CO to a larger or smaller extent. At 790 °C, only 3% of CO was found in the off gas. Since no hydrogen was detected and no solid carbon produced, the Boudouard reaction cannot be the source of this CO. A direct reaction between CO_2_ and methane that does not produce hydrogen is a possible explanation, like for example the following reaction, which is thermodynamically favourable at 790 °C:

$$\raise.5ex\hbox{$\scriptstyle 1$}\kern-.1em/ \kern-.15em\lower.25ex\hbox{$\scriptstyle 4$} {\text{ CH}}_{{4}} + \, \raise.5ex\hbox{$\scriptstyle 3$}\kern-.1em/ \kern-.15em\lower.25ex\hbox{$\scriptstyle 4$} {\text{ CO}}_{{2}} \leftarrow \to \raise.5ex\hbox{$\scriptstyle 1$}\kern-.1em/ \kern-.15em\lower.25ex\hbox{$\scriptstyle 2$} {\text{ H}}_{{2}} {\text{O }} + {\text{ CO}}$$

At 1000 °C, there was between 18 and 30% of CO, and the CO_2_ level had dropped below 10%. At 1100 °C, basically all CO_2_ had been converted.

The experiments with CO_2_ also had less hydrogen in the off-gas than when no CO_2_ was present. It is not possible, within the uncertainties, to determine to what extent this is because of methane dilution or if reactions with CO_2_ plays a role. On the one hand, the presence of CO in the off-gas indicates that CO_2_ reacts with something, which could be the produced H_2_. On the other hand, it is theoretically possible that all CO is produced from reactions between CO_2_ and deposited C, which should not influence the H_2_-levels.

While CO_2_ in the process gas giving less hydrogen in the off-gas is a drawback in isolation, there could also be an upside to having CO in the off-gas. Syngas (H_2_-CO-mixture) production from biogas without CO_2_-emissions requires the upgrading (CO_2_-removal) of biogas to biomethane, followed by storage/utilisation of the CO_2_ and steam reforming of the methane into syngas. It is possible that a competitive option would be to use non-upgraded biogas as a solid carbon source as described here, avoiding the need for CO_2_-removal and steam reforming.

The observations that temperature is a very important parameter, and that methane decomposition increases with temperature is in line with experiences from similar work^20,21^, as is the result that the degree of methane decomposition is higher in the case of a lower total gas flow^22^.

### Carbon deposition

**Figure 4:**
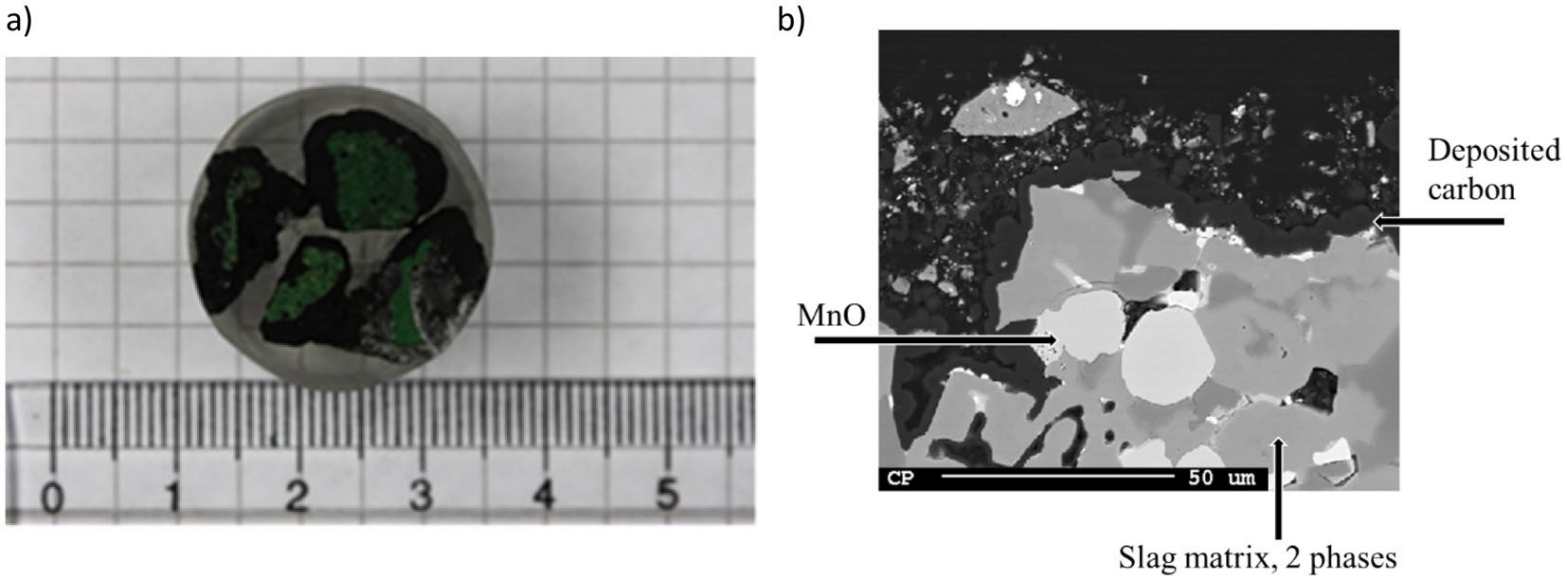
Example images of samples showing deposited carbon, both from experiment 6 (1000 °C, 90%CH_4_–10% Ar, 2.35 L/min). (**a**) Macroscopic image showing black layers on the greenish slag. (**b**) ×500 magnification back scatter EPMA image showing the different slag-phases and the deposited carbon layer. The phases were verified by X-ray diffraction.

The decomposition of methane is only the first step; the formed carbon needs to deposit onto the HCFeMn slag. Several techniques were used to verify the deposition of carbon and determine the amount deposited. The chemical analyses turned out not to be very useful for quantification since the slag contained inhomogeneously distributed carbon (likely in metallic droplets). Visual inspection of samples by eye was sufficient to identify that some samples turned completely black, suggesting carbon deposition. EPMA images showed carbon layers (verified by X-ray diffraction) on several of the samples. Example images are shown in Fig. 4.

**Figure 5:**
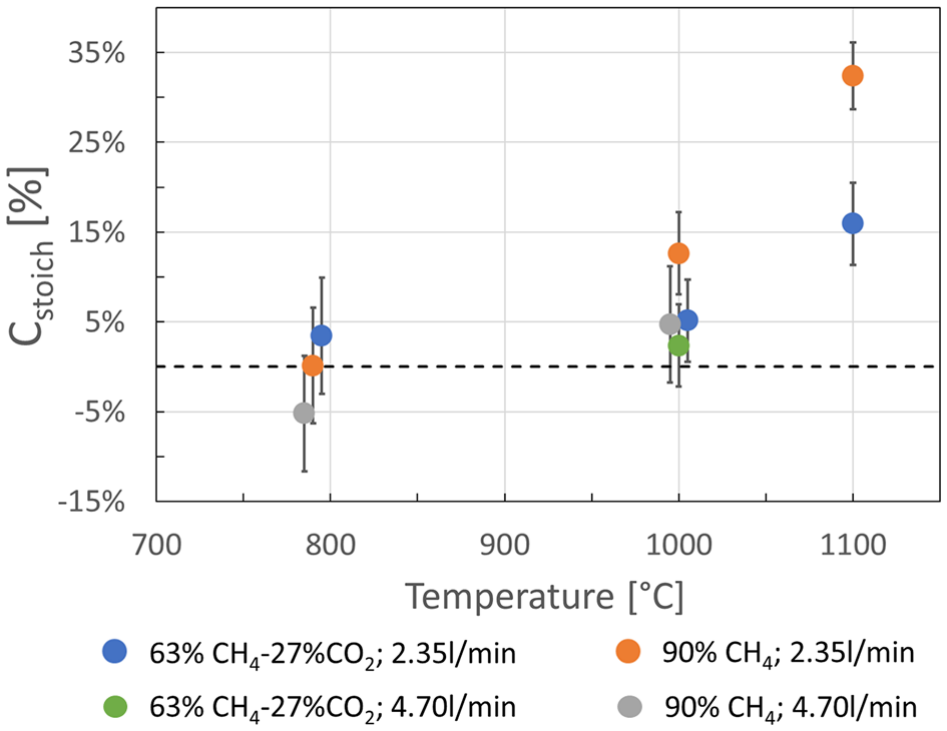
Amount of carbon deposited for all experiments, based on weight measurements, expressed as *C*_*stoich*_ (see text). For repeat experiments, each data point represents an average of all experiments with identical experimental parameters. The data points at common temperatures have been spaced out along the x-axis to improve readability. All data points have been recorded at T = 790 °C, 1000 °C or 1100 °C, respectively.

The weight change measurements are seen as the most trustworthy estimates of amounts of deposited carbon. Figure 5 shows the results with uncertainties for all experiments, where repeat experiments have been grouped and averaged. The best results are with 90% CH_4_ and 2.35 L/min total gas flow at 1100 °C, where an average of 46 g of carbon has been deposited, corresponding to 38 ± 6% of C_stoich_. In Fig. 5, the results are plotted versus temperature. The first observation that can be made is that there is no significant carbon deposition at 790 °C, an observation that matches the visual appearance of the material, as well as the absence of hydrogen in the off-gas for these experiments (Fig. 3).

For experiments with low gas flow (2.35L/min), there was significant weight change at both 1000 °C and 1100 °C, and the weight change at 1100 °C was significantly higher than at 1000 °C. This was true for experiments with and without CO_2_ in the process gas. Increased temperature thus leads to increased carbon deposition rate.

At 1000 °C, the average results with high gas flow (4.7 L/min) lie below the average results of experiments with low gas flow (2.35 L/min) for otherwise identical parameters. Since the uncertainty intervals of the low- and high gas flow experiments overlap for each set of parameters, it cannot be concluded that this is a statistically significant trend. While the gas measurements showed less methane decomposition at higher gas flow, this alone should not give an expectation of less carbon deposited, since it is possible that a reduction in decomposition rate is outweighed by an increase in total amount of methane passing through the furnace. The picture remains unclear.

The final process parameter that can be discussed in terms of its influence on the carbon deposition is the process gas composition. At low gas flows (2.35 L/min) there was deposition at 1000 °C and 1100 °C both with and without CO_2_ in the process gas. This demonstrates that carbon deposition from CO_2_-containing gas is possible. At 1000 °C and 2.35 L/min, the average deposited values are higher when there is no CO_2_ in the process gas, but since the uncertainty intervals overlap the result is not significant. At 1100 °C however, the result is much more pronounced and clearly significant. CO_2_ is expected to have two potentially negative impacts on carbon deposition in this temperature range: The first is the simple dilution of methane, meaning that less carbon is available to deposit. The second is the potential consumption of already deposited carbon through the Boudouard reaction. The indicated negative impact of CO_2_ also at 1000 °C is thus expected to be valid. However, at present it is not possible to say whether this difference is due simply to the dilution of methane, or whether chemical reactions involving CO_2_ also plays a role.

### Discussion of catalytic effects

Even though no catalyst was consciously added to the system, there may be catalytic effects at play. As mentioned in the introduction, both MnO and metallic iron have the potential to interact catalytically with the gaseous species in the system. As the goal of the current work is to deposit as much carbon as possible onto the slag, in a successful process such an effect would only play a role during the initiation of the deposition. Once the initial layer of carbon is formed, further deposition will progress as carbon-on-carbon deposition, masking any effect of the now covered slag surface.

It is still possible that the catalytic nature of the surface plays a significant role even if it directly interacts with the gas-phase only during initiation. As an example, consider the hypothesis that deposition on both metallic iron and on already deposited carbon is more favourable than deposition on a metal-free slag surface. Under this hypothesis, it can be imagined that there is a temperature range in which deposition on the pure slag is unfavourable, but it is favourable on carbon and iron. At these conditions, a slag with no iron would see no carbon deposition, while for a slag with iron impurities deposition could be initiated from metallic iron and then proceed as carbon-on-carbon deposition.

Since all industrially produced slag will have iron inclusions, the practical relevance for any real process may be cast into doubt. But if the amount and form of the iron is significant, then it can be conceived that part of the optimisation of the entire value chain (ferromanganese production, carbon deposition, silicomanganese production) would involve modifying the ferromanganese production to produce a slag with the best possible deposition properties. This could be an interesting idea to pursue once the whole concept of manganese alloy production from bio-gas has been proven. Such an undertaking would likely require the use of synthetic slags (since all industrially sourced slags contain iron).

### Abrasion strength of carbon layer

**Figure 6:**
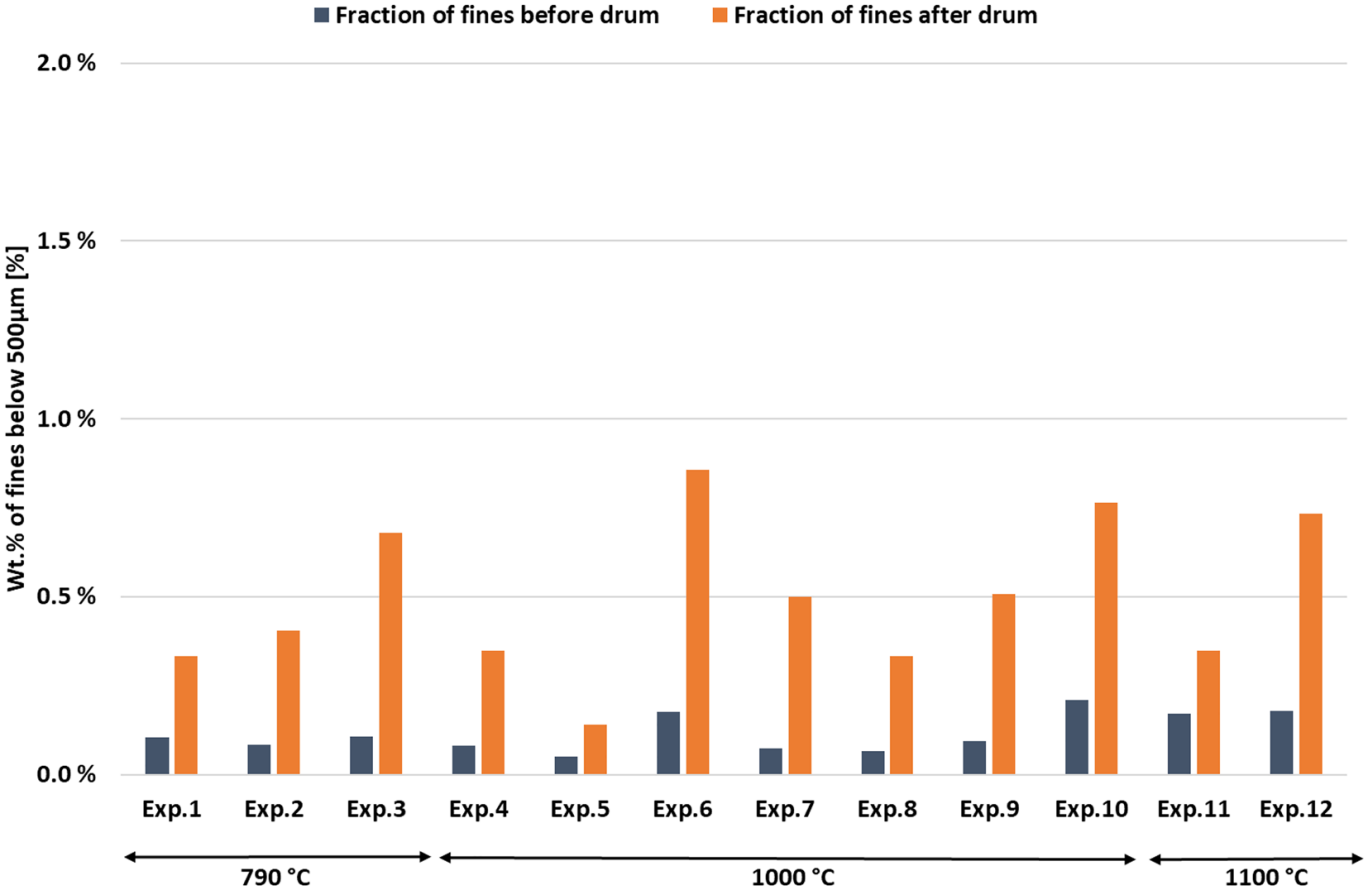
Fraction of fines (< 500 µm) before and after abrasion treatment in Hannover drum for alle experiments in experimental series 1.

Figure 6 below shows the fraction (Wt.%) of material below 500 µm before and after the abrasion test for all experiments in the first series of experiments. In all cases, treatment in the drum increases the amount of fines. It can be noted that the fines-generation in the experiments at 790 °C is comparable to that of the other samples. If the carbon layer had disintegrated easily and created a lot of fines, it would be expected that there was a big difference between samples from 790 °C and the other experiments, since the samples at 790 °C had no methane decomposition and no carbon deposited.

The generated fines-fraction was analysed for its carbon content. Where pre-drum analyses were available, an average of 99% of C remained in the > 500 µm fraction and 96% in the > 5 mm fraction. As it was mentioned earlier in the section, the Leco analysis is not the best method of comparing carbon levels in this work. As worst-case scenarios, it can be assumed that the generated fines are 100% carbon. In this scenario, for samples with significant carbon deposition, 81% of the deposited carbon material would remain in the > 500 µm fraction. When also considering the 500 µm to 5 mm fraction, the weight of material < 5 mm is greater than the weight of the deposited carbon. However, this worst-case scenario is not realistic. As the comparison of fines-generation in carbon-free materials shows, the fines fractions are far from 100% carbon.

These results point towards good adhesion between the carbon and the slag. There is nothing in these results that indicate that the material would not survive normal transport to/around an industrial plant.

### Estimation of accuracy

As was mentioned, the chemical analyses were not judged to be trustworthy estimates of the deposited carbon, and weight change data was used. However, loss of material during transfer, and dust/flakes of crucible material falling into the charge could also lead to weight change, giving rise to uncertainties in these estimates. The uncertainties can be estimated from the experiments at 790 °C, where the expected weight change is 0 but the recorded values are −7.9 g, + 4.9 g and + 0.2 g. This means that uncertainties are at least ± 7.9 g, and in this work, a value of ± 8 g has been used.

## Conclusions and outlook

This work has shown the potential for use of biogas/biomethane as a non-fossil source of carbon in manganese production, with hydrogen and/or syngas as a potential by-product.

It has been shown that carbon can be deposited on HCFeMn slag from CH_4_ and CH_4_–CO_2_ gas mixtures. While the process has not been optimised, the best achieved results gave an amount of deposited carbon equal to 37 ± 4% of the amount necessary for reduction of all MnO in the slag. This result was obtained with 90%CH4–10%Ar at 1100 °C and with 2.35 L/min total gas flow.

Temperature was the most important parameter. No CH_4_-decomposition or carbon deposition was detected below 1000 °C, and increasing the temperature from 1000 to 1100 °C gave increased CH_4_-decomposition and carbon deposition. For other parameters, a lower gas flow gave more CH_4_-decomposition, but it could not be determined whether this translated into a greater deposition of carbon. The presence of CO_2_ reduced the carbon deposition, but it is not clear at present whether it is because of dilution of methane or other effects like Boudouard reaction.

The process produces hydrogen-rich off-gas as a by-product, more than 70% in the best cases. CO_2_ in the process gas was converted into CO, and while its presence decreased the carbon deposition it did not fully supress it. It is thus possible to use non-enriched biogas as the carbon source, in which case the gaseous by-product is a mixture of H_2_ and CO. Which of these options will be the optimal solution is far too early to tell, and will not at least depend on the performance of the composite material in silicomanganese production.

## Data Availability

The datasets used and/or analysed during the current study available from the corresponding author on reasonable request.
